# Bioactive Nutrients and Nutrigenomics in Age-Related Diseases

**DOI:** 10.3390/molecules22010105

**Published:** 2017-01-08

**Authors:** Tania Rescigno, Luigina Micolucci, Mario F. Tecce, Anna Capasso

**Affiliations:** 1 Department of Pharmacy, University of Salerno, Fisciano 84084, Italy; trescigno@unisa.it (T.R.); tecce@unisa.it (M.F.T.); 2 Computational Pathology Unit, Department of Clinical and Molecular Sciences, Università Politecnica delle Marche, Ancona 60120, Italy; l.micolucci@univpm.it; 3 Laboratory of Experimental Pathology, Department of Clinical and Molecular Sciences, Università Politecnica delle Marche, Ancona 60120, Italy

**Keywords:** aging, bioactive nutrients, dietary, nutrigenomics, oxi-inflamm-aging, molecular pathological epidemiology

## Abstract

The increased life expectancy and the expansion of the elderly population are stimulating research into aging. Aging may be viewed as a multifactorial process that results from the interaction of genetic and environmental factors, which include lifestyle. Human molecular processes are influenced by physiological pathways as well as exogenous factors, which include the diet. Dietary components have substantive effects on metabolic health; for instance, bioactive molecules capable of selectively modulating specific metabolic pathways affect the development/progression of cardiovascular and neoplastic disease. As bioactive nutrients are increasingly identified, their clinical and molecular chemopreventive effects are being characterized and systematic analyses encompassing the “omics” technologies (transcriptomics, proteomics and metabolomics) are being conducted to explore their action. The evolving field of molecular pathological epidemiology has unique strength to investigate the effects of dietary and lifestyle exposure on clinical outcomes. The mounting body of knowledge regarding diet-related health status and disease risk is expected to lead in the near future to the development of improved diagnostic procedures and therapeutic strategies targeting processes relevant to nutrition. The state of the art of aging and nutrigenomics research and the molecular mechanisms underlying the beneficial effects of bioactive nutrients on the main aging-related disorders are reviewed herein.

## 1. Introduction

Aging can be viewed as a multifactorial process stemming from the interaction of genetic and environmental factors, which include lifestyle. It is characterized by the onset of several age-related diseases (ARDs) such as dementia, osteoporosis, arthritis, diabetes, cardiovascular diseases (CVDs), neurodegenerative disorders, and cancer which, though not unique to old age, are nonetheless closely related to it. The physiological decline experienced by organisms over time is a key factor in increasing the risk of developing ARDs [1,2]. As human life expectancy expands, the number of patients with ARDs is rapidly increasing and will continue to mount, posing a serious challenge to healthcare systems globally [3]. ARDs are also becoming a key social and economic problem [4]. Progress in the understanding of genetic associations, particularly via genome wide association studies, has disclosed a substantial contribution of genes to human aging and ARDs [4].

Ample evidence from several species indicates that the maximum age attainable is genetically determined and that multiple mitochondrial DNA polymorphisms are associated with longevity [5]. The several theories of aging that have been devised over the past few decades have failed to provide an answer to the questions: “why do we age?”, “what can we do to live longer?”. However, the notion of aging as a complex multifactorial process has superseded previous constructs based on single factors [6]. In fact, whereas some of the processes that characterize physiological aging can be explained by individual factors, no single theory can account for aging as a process. Several different molecular mechanisms linking aging and ARDs have been advanced [3].

Various lines of research have demonstrated that telomerase activity and telomere length shortening play important roles in aging, Alzheimer’s disease (AD) and type 2 diabetes (T2DM) [7,8]; a reduced capacity for DNA repair and genomic instability are commonly seen in both aging and cancer [4,9,10]; mitochondrial dysfunction is a hallmark of aging and ARDs, including CVDs and cancer [11,12]; and metabolic syndrome, diabetes, CVD, neurodegenerative diseases, and other ARDs are associated with chronic inflammation [13,14,15].

In fact, most of the phenotypic characteristics of aging are the result of an age-related, low-grade, chronic proinflammatory status that has been designated “inflammaging” [16], which is partly under genetic control. Moreover, up-regulation of inflammatory responses induces senescence, and inflammatory changes are shared by several age-related diseases. Oxidation-inflammation has thus been hypothesized to be the main cause of aging (oxi-inflamm-aging) [17]. The aging-related chronic oxidative stress affects all cells, especially those of regulatory systems (nervous, endocrine and immune systems) and the communication among them, adversely affecting homeostasis and the maintenance of the health status. Since the redox state and functional capacity of immune cells are related to longevity, the immune system is also likely to be critically involved in aging and to affect its rate. Moreover, the role of the immune system in senescence is likely to be pervasive, as also confirmed by the demonstration that adequate dietary antioxidants improve immune function, reduce oxidative stress, and increase longevity [18].

Human molecular processes are influenced by physiological pathways as well as exogenous factors, including dietary components. Since nutrients directly affect physiological changes, the diet has substantive effects. For instance, bioactive molecules capable of selective modulation of specific metabolic pathways affect the development/progression of cardiovascular and neoplastic disease. As bioactive nutrients are increasingly identified, their clinical and molecular chemopreventive effects are characterized and systematic analyses encompassing all “omics” technologies (transcriptomics, proteomics and metabolomics) are conducted to investigate their effects. Nutrigenomic knowledge regarding physiological status and disease risk is expected to lead to the development of improved diagnostic procedures and of therapeutic strategies targeting processes related to nutrition.

The state of the art of aging and nutrigenomics research and the molecular mechanisms underlying the beneficial effects exerted by bioactive nutrients on the main ARDs are reviewed herein.

## 2. Aging and Age-Related Diseases

### 2.1. Aging Theories

A number of theories have been proposed to account for the unavoidable effects of aging. The main aging theories are the genetic theory, cellular aging theory, the neuroendocrine theory, the immunological theory, the free-radical theory, and the network theory.

The *genetic theory* [2] suggests that aging is the direct consequence of a genetic programme, and that the lifespan of each animal species is regulated by genetic factors. Indeed, in the human species the members of some families are particularly long-lived and diseases such as Down, Werner, and Hutchinson-Gilford syndrome, which are characterized by an accelerated aging process and a shortened lifespan, are hereditary [19,20]. Some studies do not focus on “gerontogenes”, i.e., genes that actively determine aging, but rather on “longevity assurance genes”, genes that guarantee a long life. Extreme longevity appears to be related to a complex genetic pattern rather than to a few isolated genes [19,20].

According to the *cellular theory*, cellular senescence limits the number of divisions that normal human cells can undergo in culture [21]. Renowned experiments conducted by Hayflick have demonstrated that embryo fibroblasts from a variety of animal species can replicate only a finite number of times in proportion to the lifespan of the individuals of the species [21]. The Hayflick limit is reached after a given number of cell divisions [22,23,24,25] or due to a reaction to given molecular events. Replicative senescence, which correlates to the number of cell divisions determined by telomere length, can therefore be considered as one of the causes of aging, whereas stress-induced senescence is a response to a sudden modification of the genome and to DNA damage. Cellular senescence should therefore be viewed as a response to age-related changes, which accelerate the organism’s aging process. This view of senescence is consistent with the theories of damage build-up (like the free-radical theory), which may explain the ultimate cause of cell senescence through aging [26].

The hypothesis underpinning the *neuroendocrine theory* of aging is that aging results from the dysregulation of the neuroendocrine system by chronic exposure to physical, biological, or emotional stress, which may overburden or damage the organism’s adaptive capacity, leading to “adaptation diseases” and ultimately death [27,28,29,30,31]. The integration of such stimuli seems to be performed by the hypothalamus through information provided by a variety of brain structures, chiefly the cerebral cortex, the limbic lobe, and the reticular formation. The hypothalamic signals induce production and secretion of pituitary hormones, which regulate key body functions, and stimulate peripheral endocrine glands (e.g., adrenal glands, thyroid, and gonads), whose hormone products are conveyed to their remote targets through the circulation [26]. In this highly sensitive, closed-circuit system, hormone production is modulated to meet the changing needs of the body; these are induced by continuously changing stimuli, which are stressful in the largest sense of the word, encompassing for instance heat-cold, food-fast, activity-rest, and sleep-vigil. Over time, the mechanism may undergo functional alterations and bring about irreversible modifications in all organs and apparatuses [32,33,34,35,36,37,38,39,40,41,42].

The *immunological theory* of aging holds that physiological aging is related to immune system dysregulation [43]. The term “immunosenescence” has been proposed to describe the aging-related impairment of immunocompetence, which makes the elderly more susceptible to disease and increases morbidity and mortality from diseases linked to infectious agents via impairment of the response to acute infection as well as vaccines. According to some researchers, immunosenescence also involves an increased susceptibility to cancer and a diminished capacity of self-recognition, which lead to autoimmune disorders [44].

The best known age-related changes affecting the immune system include: (i) thymus involution, resulting in decreased production of T cells, which are responsible for acquired and cell-mediated immunity [45]; (ii) lymphocyte remodelling, which is closely related to the T-cell compartment [46,47,48,49,50,51,52,53,54,55,56,57,58]; and (iii) changes in the secretion pattern of pro- and anti-inflammatory cytokines [16,59,60,61,62,63,64,65,66,67,68,69].

The *free radical theory* rests on the evidence that aerobes produce oxygen-centred free radicals which irreversibly damage biological structures. Free radicals are formed inside cells as a result of the participation of oxygen in energy. Mitochondrial respiration generates reactive oxygen species (ROS) through leakage of intermediates from the electron transport chain [70]. ROS are highly unstable molecules, due to an unpaired electron, and strive to attain a stable state by appropriating electrons from nearby molecules, which in turn become unstable, thus propagating the instability.

The *network theory*, formulated in 1989 by Franceschi and colleagues [71], unifies a number of previous theories [72]. This constructs accepts that aging depends on genetic and environmental factors. The organism is exposed to endogenous and exogenous noxious agents that are physical (radiation, heat), chemical (toxic metabolites, free radicals), and biological (viruses and pathogenic microorganisms). The main mechanisms activated by the organism to preserve homeostasis include DNA repair, stimulation of the antioxidant system, production of anti-inflammatory cytokines and heat shock proteins, activation of poly-ADP-ribose polymerase (a DNA-repairing nuclear enzyme), and apoptosis, which is considered as the ancestral process by which damaged, mutated, infected, or transformed cells are removed [54,56]. All these cellular and molecular processes have become layered throughout evolution [56].

### 2.2. Inflammaging

The term inflammaging, introduced by Franceschi [16], indicates that aging is characterized by a low-level, chronic, asymptomatic, systemic inflammatory state involving the body’s adaptive systems in a sustained effort to provide a myriad, low-grade inflammatory responses that tend to become chronic and are frequently asymptomatic [54,73,74,75,76,77,78]. It has been shown that this type of chronic cell stress can induce the acquisition of the senescence-associated secretory phenotype (SASP), which is characterized by the activation of a proinflammatory transcriptional programme [79,80]. Patients report vague and non-specific signs and systemic symptoms that are difficult to organize into a diagnosis. Inflammaging is thus the result of the body’s ability to adapt and react to the effects of a variety of stress factors that induce the accumulation of molecular and cellular damage.

#### Physiopathogenesis

The relationship between chronic systemic inflammation and aging is widely accepted. Strong evidence indicates that most of the phenotypic characteristics of the aging process are induced by inflammaging, which is partly under genetic control and which results from continuous antigenic stimulation that continues after reproductive age, a phenomenon for which evolution has not provided [67,81,82]. The consequent cellular damage appears to increase the risk of death among the elderly and to affect longevity adversely [83]. A number of genetic, cellular, and serological markers of inflammaging have been identified, such as an immunophenotype characterized by a reduction in naive T cells and an accumulation of memory cells, increased levels of proinflammatory cytokines, and significant alterations in the frequency of functional pro- or anti-inflammatory polymorphisms [69,73,84]. Inflammaging is characterized by macrophage activation and by expansion of specific T-cell clones (megaclones) directed to common virus antigens such as cytomegalovirus (CMV) and Epstein-Barr virus [85,86,87,88,89,90]. In a study of 121 individuals aged 25 to 100 years, 18 CMV-negative (−) and 103 CMV-positive (+), CMV+ subjects exhibited an accelerated age-related reduction of naive CD8+ T cells as well as a progressive increase in CD28− and CD8+ effector T cells [90]. Therefore, CMV seropositivity seems to be associated with multiple phenotypic and functional alterations of T-cell immunity that are considered as biomarkers of aging [90]. Herpes simplex virus (HSV) infection is another chronic infection that may affect the immune/inflammatory response in the elderly and that appears to be a cofactor in the damage produced by AD [91,92,93].

The complex inflammatory status that is the hallmark of inflammaging is mainly related to the increase in circulating proinflammatory cytokines. Cytokines are a class of soluble proteins that are responsible for the communication among the different immune system components. They play an important role in inflammation by acting on the targeting, regulation, and termination of inflammatory processes, and also participate in the aging process. Aging involves a reversible decrease in interleukin (IL)-2, a cytokine that has a role in the development of Th1 populations and in increased production of proinflammatory mediators such as IL-1, IL-6, and tumour necrosis factor (TNF) α [56]. The increase in circulating age-related inflammatory markers could underpin the reduced ability of elderly organisms to cope with various stressors. Inflammaging may also generate ROS that cause oxidative damage and induce an increase in cytokine release, fuelling a vicious circle where tissue damage and repair mechanisms are simultaneously activated, giving rise to a chronic proinflammatory state. The damage accumulates slowly and asymptomatically over decades, resulting in aging and ARD development [54,74,75,76,77,78]. ROS are also capable of exerting strong effects on gene expression and are implicated in the pathogenesis of numerous ARDs such as atherosclerosis, T2DM, neurodegenerative disorders, osteoporosis, and osteoarthritis, which all share a strong inflammatory/immunological component. Moreover, oxidative stress and ROS induce apoptosis and may act as mediators, influencing other transcription factors, like NF-κB and AP-1 [73]. Aging-related alterations in apoptosis may thus account for some prominent features of immunosenescence [82], such as the accumulation of memory cells, megaclone expansion, the reduced T-lymphocyte repertoire, and the increased incidence of autoimmune disorders.

Moreover, emerging evidence suggests that DNA damage response (DDR) signalling is a key mechanism linking the build-up of DNA damage, cell senescence, and organism aging [94]. This evidence suggests the involvement of epigenetic modifications; for instance microRNAs, small, non-coding RNAs involved in post-transcriptional regulation, have been hypothesized to play a key role in the diffusion of DDR/SASP signalling to non-damaged surrounding cells during aging, suggesting that the identification of new DDR/SASP signalling components may enable the development of novel therapeutic interventions against ARDs [9]. The hypothesis has also been advanced that microRNAs may be harnessed as innovative tools to detect and target senescent cells and to develop therapeutic interventions that can slow down the proinflammatory programme activated in senescent endothelial cells [95].

Conversely, the cellular and molecular mechanisms related to the body’s ability to respond to chronic oxidative stress and inflammation appear to play an important role in promoting longevity and in avoiding/delaying the major ARDs. A role for inflammatory cells and molecules in the pathogenesis of ARDs such as atherosclerosis, AD, and Parkinson’s disease has clearly been documented. The control of inflammation may be capable of fostering successful aging. That this goal can be achieved is demonstrated by centenarians, living examples of successful aging who have attained the desirable form of aging, because they do not suffer from chronic debilitating diseases, they are physically self-sufficient, and have preserved their cognitive abilities. The study of centenarians has the potential to provide insight into the biological basis of healthy aging or on the combination of genes and lifestyle that can prevent the major ARDs. The INCHIANTI study, which ended in 2004 [96], showed that healthy elderly subjects have high levels of IL-6, IL-1 and C-reactive protein (CRP) compared with healthy young subjects. A further study of a group of Italian centenarians has shown that individuals genetically predisposed to produce IL-6 in old age are less likely to reach the extreme boundaries of human lifespan.

Lio and colleagues [76] have assessed the levels of two cytokines, the anti-inflammatory IL-10 and the proinflammatory TNF α, in a group of centenarians and found that compared with younger subjects they express genes coding for high levels of IL-10 and low levels of TNF α. Moreover, the frequency of the variants (polymorphisms) of key genes involved in immune response and low-grade inflammation are found with different frequencies in centenarians and young individuals [89,97,98]. The identification of an anti-inflammatory genotype in centenarians suggests that chronic inflammation is a key predictive marker of mortality/morbidity.

Evidence of inflammaging has also been found in healthy centenarians, who exhibited up-regulation of markers such as the anti-inflammatoryIL-10 and the proinflammatory TGF β [99,100]; these data suggest that anti-inflammaging activity is also present in these subjects, and is equally important for longevity, and that longevity is the result of the balancing of such conflicting processes [101]. A lifespan exceeding 90 years seems to have a strong genetic basis, explaining why the almost-100-year olds and centenarians tend to belong to the same families. Longevity seems to be influenced by a complex genetic pattern, not by a few isolated genes, suggesting selection for genes and genetic variants associated with strong immune responses and inflammation.

The hypothesis has been advanced [88,102] that an effective inflammatory response directed at combating infection at a young age may eventually be the cause of conditions such as arthritis, diabetes, CVD, and neurodegenerative disorders in old age. The dual biological role of inflammation, positive at a young age and negative in old age, is consistent with antagonistic pleiotropy theory, whereby a gene can exert opposite effects in different periods of life. Inflammation is not a negative phenomenon per se; indeed, in responding to various stimuli, the immune system enacts a complex series of local and systemic reactions that limit tissue damage, isolate and destroy infectious agents, and activate repair processes. Its adverse effects are related to the increased life expectancy, which has not been provided for by evolution. An extended lifespan involves that the immune system continues to react against external agents for decades longer than it has been programmed for, and that such increased antigenic load eventually establishes a chronic inflammation that contributes to the deterioration of various organs, becoming a risk factor for all ARDs. Elderly individuals with higher blood levels of an acute-phase protein, CRP, are especially prone to chronic inflammatory diseases.

The systemic consequences of inflammaging are associated with a pattern of changes that has been designated “frailty” [101]. Epidemiological studies suggest that such changes promote an atherogenic profile that is shared by other chronic inflammatory ARDs. Other genetic and environmental factors that promote disease continue to exert their effects and even determine the main organ that will be affected. Differences in inflammatory status partly explain why not all elderly subjects who share the same risk factors go on to develop ARDs. The genetically determined immune system potential is therefore gradually depleted over time. Improvements in hygienic conditions may have reduced significantly the antigenic overload, delaying the depletion. This factor, besides the reduced mortality from acute infectious diseases, may contribute to the increased life expectancy and to the increase in the number of subjects who reach the extreme boundaries of the human lifespan.

### 2.3. Aging-Related Disorders

Inflammation is a chronic, systemic cause of several ARDs, including atherosclerosis, CVD, AD and cancer. Recent data show that chronic systemic inflammation contributes to anxiety, depression, cognitive decline, insulin resistance and adult-onset diabetes, obesity, and Parkinson’s disease.

#### 2.3.1. Cancer

Cancer mostly affects elderly subjects. In industrialized countries, average age at cancer diagnosis is close to 70 years, and is expected to increase [103]. Prolonged exposure to carcinogenic factors, increased cell susceptibility to environmental carcinogens [104,105], and immunosenescence [106] are believed to be major reasons for the predominance of cancer in the later decades of life. A strong link between the chronic inflammation induced by chemical, biological, mechanical, or physical lesions and cancer is well documented. For example, bowel inflammatory disease, ulcerative colitis, and Crohn’s disease predispose to the development of cancer of the large intestine or terminal ileum [107,108] and *Helicobacter pylori* infection is associated with atrophic gastritis, mucosal dysplasia, and gastric adenocarcinoma. Inflammation is also involved in the development of solid tumours such as colon cancer, as demonstrated by a prospective case-control study where the 172 subjects (out of 22,887 adults who were followed for 11 years) who developed colon cancer had higher plasma CRP [109]. Cancer susceptibility and severity may be associated with functional polymorphisms of cytokine genes involved in regulating inflammation; in particular, polymorphisms of IL-6 and IL-10 genes may influence cancer susceptibility and in some cases its prognosis.

A variety of different mechanisms may link inflammation to cancer, since (i) induction of angiogenesis by inflammatory factors promotes cancer progression [110]; (ii) increased release of proinflammatory factors and certain cytokines such as IL-1, TNF α and interferon are involved in inflammation and cancer development [110,111]; (iii) free-radical production promotes carcinogenesis [110].

The inflammatory state is a key factor in the intermediate stages of tumour development. In cancer, genetic damage triggers disease onset and inflammation fuels the process. In 1978, Alberto Mantovani found that innate immunity cells tend to cluster around some tumours [112]. Pollard and colleagues subsequently showed that cancer cells “re-educate” macrophages, turning them into cytokine and growth factor factories that stimulate cancer growth by acting as tumour promoters [112]. Macrophages produce TNF, which activates nuclear factor NF-κB in cancer cells, triggering the production of proteins that stop apoptosis and activate cell proliferation. The innate immune system is thus harnessed to help the tumour grow. How the process starts is, however, still unclear [112]. Interestingly, cancer development is largely avoided or delayed in centenarians, where changes in some specific microRNAs have been detected in plasma and leukocytes [113].

#### 2.3.2. Atherosclerosis

Almost 50% of all deaths in the developed world and 25% of those occurring in developing countries are related to CVD. Atherosclerosis is the leading cause of heart disease and stroke. Atherosclerosis, which used to be considered as a disease of lipid accumulation, is now viewed as a chronic inflammatory disorder affecting large and medium-sized vessels [114]. Lesions arise in childhood as reversible lipid streaks which in old age tend to become plaques that may reduce the arterial lumen, or else become ulcerated and give rise to thrombosis that may result in lumen occlusion. Clinical manifestations range from angina pectoris and myocardial infarction when the coronary arteries are involved, stroke if it is the arteries of the central nervous system and peripheral arterial disease if it is the peripheral circulation. Vessel branches and bends are more prone to host atherosclerotic lesions, due to surface friction (hemodynamic stress), an important factor in intima thickening. Early atherosclerotic lesions are identified by the dysfunction induced by cardiovascular risk factors (smoking, hypercholesterolaemia, hyperhomocysteinaemia, hypertension, obesity and diabetes mellitus, and possibly infectious and immunological causes) and by the accumulation and subsequent oxidation of low-density lipoprotein (LDL). Endothelial dysfunction evolves to a situation characterized by monocyte and T-cell migration and adhesion to the intima in response to the surface expression of endothelial adhesion molecules, e.g., selectins, VCAM-1, ICAM-1 and chemotactic signals (e.g., MCP-1). The monocytes recruited to the intima proliferate and differentiate into macrophages that phagocytose oxidized lipoproteins, turning them into foam cells that characterize fatty streaks. Secretion of cytokines and growth factors, mainly from macrophages, induces migration of smooth muscle cells from the media to the intima and their proliferation and differentiation into a phenotype that synthesizes extracellular matrix. As a result, lipid streaks are transformed into advanced lesions, i.e., fibrous plaques formed by a fibrous cap that encloses a lipid core. LDL accumulation is due not only to the increased permeability of the functionally damaged endothelium, but also to its ability to bind the extracellular matrix constituents in the intima. LDLs undergo oxidation and are then trapped in the extracellular matrix of the subendothelial space where they play a key role in the development of the chronic intimal inflammation. LDLs are oxidized by enzymes and oxidative metabolites produced by arterial wall cells, especially monocytes-macrophages recruited in the intima. In this context, they activate some transcription factors (e.g., NF-κB) in cells (endothelium, macrophages, smooth muscle cells), which induce the expression of genes encoding adhesion molecules, cytokines, and growth factors and trigger the inflammatory response, activate platelets and cause aggregation. Fibrous plaques that develop ulceration, bleeding, thrombosis, and calcification, lead to the third and most severe atherosclerotic stage: complicated lesions. Plaque fissuring is held to be the result of several factors, particularly plaque inflammation and an abundant lipid component, which would make the plaque less resistant to the blood component. Inflammatory cells and especially macrophages produce hydrolytic enzymes such as metalloproteases that can lyse the collagen of the fibrous cap, impairing its resistance to hemodynamic stress.

#### 2.3.3. Alzheimer’s Disease

AD is the most common neurodegenerative disease in the West. The aging of the population is making it an increasingly severe public health problem. The disease manifests with a progressive decline in memory and intellectual abilities, impoverishment of language and behavioural skills, and disorientation. Characteristic neuropathological features are senile plaques (SPs), neurofibrillary tangles (NFTs) and amyloid angiopathy. NFTs are accumulations of dystrophic neurites containing double helix filaments whose main component is the phosphorylated form of τ protein encoded by chromosome 17 and associated with microtubules. β amyloid deposits are seen both in brain vessel walls and, more typically, in SPs, which consist of a central core of β amyloid fibrils surrounded by a ring of dystrophic neurites, reactive astrocytes and microglia. The β amyloid protein originates from the cleavage of a precursor consisting of two fragments of 40 (Aβ1-40) and 42 amino acids (Aβ1-42). In the familial form of AD, more than 50% of patients bear mutations in the amyloid precursor protein on chromosome 21 [115], presenilin 1 gene on chromosome 14 [116], and presenilin 2 gene on chromosome 1 [117]. These changes are associated with increased production of Aβ1-42, which is highly toxic for neurons and is the main SP component. However, the role of genetic factors in the pathogenesis of sporadic AD is not completely clear, and multiple risk factors are likely to be involved.

Immune responsiveness in AD appears to be altered [118,119]. Some acute-phase proteins and elements of the immune system have been detected in the brain of these patients besides the classic signs of inflammation such as oedema and neutrophil invasion. Alterations found in the brains of AD patients but not in age-matched healthy controls include a greater number of receptors for immunoglobulin and for the complement, increased microglial expression of the major histocompatibility complex, increased production of cytokines (IL-1β, IL-6), increased acute-phase proteins (plasma α 1-antichymotrypsin, α1AC) and infiltration of T lymphocytes in tissues [120,121,122,123]. A case-control study [120] showed that plasmaα1AC levels are related to the degree of cognitive impairment in AD patients and that peripheral markers of inflammation or altered immune response could be used to monitor disease progression. The role of inflammation is further stressed by epidemiological studies showing that the long-term use of non-steroidal anti-inflammatories may protect against AD [124]. Several studies have reported increased concentrations of some cytokines and their receptors, which regulate and amplify immune responses, whereas lymphocytic infiltration does not seem to play a large role. The complement system is involved in these reactive processes [125]. Its activation prepares the cell for phagocytosis and stimulates cytolysis. Antibodies against complement components have been described in brain tissue of AD patients, but they showed no binding in tissue from age-matched controls. These and other data indicate that AD involves activation of the complement cascade to produce a lytic membrane complex; neurons exposed to the complex are protected by an increased synthesis of inhibitors. However, self-destruction and phagocytosis are the predominant processes. These findings provide valuable information for AD treatment and raise questions on issues such as the relationship between β amyloid and inflammatory response. β amyloid appears to directly activate components of the inflammatory response such as the complement cascade and microglia [126] and macrophage activity.

In conclusion, the brain lesions associated with AD, such as NFTs and SPs, are characterized by a broad spectrum of inflammatory mediators produced by cells residing in the brain, including neurons. Though of secondary importance compared with the fundamental cause that determines their formation, there is strong evidence that inflammation exacerbates neuronal loss. Consequently, the AD risk is substantially influenced by several polymorphisms in the promoter region of genes, and other non-coding regions, coding for inflammatory mediators. Alleles that support the up-regulation of inflammatory mediators or that favour the down-regulation of anti-inflammatory mediators are more frequent in AD patients than in controls. Since polymorphisms are fairly common in the general population, there is a strong possibility that all individuals inherit one or more high-risk alleles [123,127,128,129,130,131,132,133,134].

## 3. Bioactive Nutrients and Nutrigenomics

The interactions among genomic and environmental factors is crucial in the development/progression of several human diseases. The diet is a key environmental factor with a prominent role in disease aetiology [135].

The diet primarily meets the metabolic and energy requirements of body composition homeostasis. However, it may also enhance health through regulation of specific processes [136], positively counteract inflammaging and the epigenetic changes associated with aging, and promote health [137,138]. Indeed, nutrients are considered as dietary signals capable of affecting both metabolic programming and cell homeostasis. Bioactive nutrients or chemopreventive molecules exert effects on human health and reduce disease risk through specific molecular mechanisms [139,140,141]; experimental and epidemiological evidence emphasizes the potential of dietary components, both macronutrients (carbohydrates, protein, fat, and fibre) and micronutrients (antioxidant vitamins and minerals) as first-line interventions in the prevention and treatment of cancer and other diseases [140,141]. Nutritional research studies span numerous disciplines and are conducted at the molecular and genetic level [142,143]. For instance, a recent review of studies into the effect of the Mediterranean diet (MD) on inflammaging, cancer, and most ARDs has found that the MD and its individual bioactive nutrients modulate several interconnected processes involved in tumorigenesis, the inflammatory response (e.g., free radical production, NF-κB activation, and the expression of inflammatory mediators), and the eicosanoid pathway. In particular, the authors highlight the evidence indicating that the MD can affect the balance between pro- and anti-inflammaging and some emerging topics, such as the maintenance of gut microbiota homeostasis and the epigenetic modulation of carcinogenesis through specific microRNAs [144]. Moreover, a parallel randomized trial has investigated the effect of a healthy diet on inflammaging and its consequences on the prevention of age-related decline in European elderly individuals [145].

The effects of food at the genetic and epigenetic level are examined by two new approaches, nutrigenetics and nutrigenomics, which assess the influence of dietary components on health and disease onset, progression, and treatment. Nutrigenetics examines how genetic variation affects the response of an organism to a given diet, evaluating the risks and benefits of specific diets and dietary components and formulating “personalized nutrition” recommendations. Nutrigenomics investigates how nutrients affect gene expression and downstream processes [146].

Nutrigenomics and nutrigenetics clearly straddle multiple research fields that span from nutrition to bioinformatics, molecular biology, genomics, functional genomics, epidemiology, epigenomics, transcriptomics, metabolomics, proteomics, lipidomics, and the microbiome [147].

To an extent, nutrigenomics approaches pharmacogenomics, which involves the systematic study of the effect of drugs on the genome [148]. However, whereas drugs are pure compounds, acting with affinity and selectivity on a limited number of biological targets through administration of precise and low doses, nutrigenomics addresses the complexity and variability of the diet, where some nutrients may be consumed in high albeit non-toxic concentrations (from µmM to mM) and may also bind to targets with different affinities and specificities [149,150].

Eating patterns influence gene, protein expression and metabolism and may thus be considered as endogenous cellular mediators. Once it is absorbed at the cell level, a nutrient is capable of interacting through specific signalling pathways, and even small changes in its structure may involve differential activation of metabolic steps. Fatty acids and their degree of carbon chain unsaturation are a valuable example, since *n-*3 polyunsaturated fatty acids promote anti-inflammatory pathways, whereas *n-*6 polyunsaturated fatty acids induce synthesis of proinflammatory molecules; in addition, *trans* fatty acids increase plasma LDL-cholesterol [151,152,153] whereas *n-*3 polyunsaturated fatty acids do not have this effect.

It is likely that several dietary compounds exert their protective and restorative action through modulation of distinct signal transduction pathways. Nutrients have been found to affect gene expression as a consequence of a direct interaction with transcription factors [154]; for instance fat-soluble ligands, such as vitamin A/D, activate their cognate nuclear receptors for ligand-dependent transcriptional regulation. Population studies based on dietary questionnaires supply useful data to relate dietary intake to phenotypes and to the risk of developing a number of diseases [155]. Moreover, actual nutritional intake can be monitored by measuring specific molecules in blood, urine, fat, and tissues, an approach that can also enable identification of the nutritional biomarkers that connect nutrition and health. Altered serum lipids (e.g., cholesterol, triglycerides), increased blood pressure, and reduced insulin sensitivity are common predictors of diet-related diseases. A broad biomarker panel, rather than a search limited to single markers, would be able to provide exhaustive information to characterize the health status of individuals.

The application of “omics” technologies to nutrition and bioinformatic data analysis allows integrating information from the closely interconnected fields of transcriptomics, proteomics, and metabolomics, to identify specific differences related to nutritional habits and to investigate the mechanisms that cause changes [147,148,156,157,158,159,160,161].

The risk of developing disease is partly under genetic control. Nutrigenetics investigates the genetic variations induced by individual nutrients, for instance by relating single nucleotide polymorphisms and point mutations in DNA sequences to diet responsiveness [162,163]; population differences in single nucleotide polymorphisms can help risk assessment and prediction, enabling formulation of lifestyle recommendations. Phenylketonuria has been the first case of a condition induced by a single gene defect to be successfully treated with a dietary, i.e., nutrigenetic, intervention, namely a low-phenylalanine diet [164].

Nutriproteomics, a recent branch of proteomics, studies protein structure and function and protein-protein interactions to identify the molecular targets of dietary components [165,166,167,168,169,170,171,172,173,174,175,176,177,178,179,180,181,182,183,184,185,186,187,188,189,190,191,192,193,194,195]. Here, too, the goal is to detect differences in protein patterns induced by given interventions, for instance a dietary treatment. In turn, proteomics analyzes the effect of dietary components at various levels, investigating peptides as bioactive markers and seeking information on nutritionally relevant biological pathways in view of the development of dietary interventions to be applied in the clinic. Proteomics, also combined with gene expression analysis, has been used in cancer prevention studies to identify novel biomarkers [196,197,198,199,200,201,202,203,204,205,206].

A quantifiable change connecting a normal or pathological condition to modulation of mRNA, a protein, or the concentration of a metabolite can be used as a molecular biomarker. In particular, a protein concentration is a practical biomarker and a valuable diagnostic tool, due to its reproducible and accurate determination [207]. The dietary levels of most nutrients are only weakly biologically active and probably have several targets. When a biomarker is tested, the timing of its response(s) should be considered according to the nutrient’s bioavailability and bioefficacy. Notably, although biomarkers may correlate with nutrient intake, their modulation may in fact be the result of a more complex process including intake, absorption, metabolism, and excretion, as well as environmental factors and genetic predisposition. Based on these considerations, successful investigation may well need a combination of proteomic biomarkers and information from other “omics” technologies.

The levels of individual metabolites can be considered as the final step in a biological process that is influenced by genetic and environmental factors, including, critically, nutritional intake.

The metabolome (from genome) is the complete set of metabolites in an organism, and metabolomics studies classify and quantify them individually in a biological fluid, cell culture, or tissue sample. Their levels, determined by analytical methods, supply information on how enzymes and the other functional proteins affect cellular homeostatic mechanisms [180]. Nutrients can interact directly with our body at the level of organs, cells, and molecules. They usually come in complex mixtures, where the amount of each compound and its interaction with multiple components are crucial, since they influence bioavailability and bioefficacy. Metabolomics allows systematic investigation of small organic molecules, and in conjunction with nutrigenomics establishes how those molecules can reflect the effects of different diets [182]. A key goal of nutritional metabolomics is to detect and identify all endogenous human metabolites and exogenous components consumed through food that are found at least transiently in human body fluids. The development of metabolite panels related to specific nutrition states would be able to characterize physiological and pathological conditions more exhaustively than dosing of a single molecule, and such information could be integrated with the data obtained with the other “omics” technologies [147,183,184,185].

A further approach that deserves to be mentioned in relation to the role of nutrients in health and disease is nutritional epigenetics, which endeavours to explore gene-diet interactions and can provide information on the role of nutrition in aging and ARD development [137,208]. Epigenetic traits are defined as heritable DNA modifications that regulate chromosome architecture and modulate gene expression, without changes in the underlying bp sequence, ultimately determining phenotype. DNA methylation and post-translational histone modifications are well-established levels of epigenetic regulation. Epigenetic phenomena are critical from embryonic life to old age, and epigenetic pattern aberrations are recognized as aetiological mechanisms in several ARDs including cancer, CVD and neurodegenerative disorders. Nutrients can act as sources of epigenetic modifications and can regulate the site where they take place. Nutrients involved in one carbon metabolism, i.e., folate, vitamin B12, vitamin B6, riboflavin, methionine, choline, and betaine, are involved in DNA methylation through modulation of the levels of the universal methyl donor *S*-adenosylmethionine and of the methyltransferase inhibitor S-adenosylhomocysteine. Other nutrients and bioactive components of food—e.g., retinoic acid, resveratrol, curcumin, sulphoraphane and tea polyphenols—affect epigenetic patterns by modulating the levels of *S*-adenosylmethionine and *S*-adenosylhomocysteine or the enzymes that catalyze DNA methylation and histone modifications. Aging and ARDs are associated with profound epigenetic changes, even though it is unclear whether such changes are programmatic or stochastic [209]. Future work in this field will need to characterize the epigenetic pattern of healthy aging, to learn which nutritional measures can contribute to maintain or achieve it.

A large number of metabolically active metabolites are produced by the microbiome, particularly the gut microbiome, which may be viewed as a complex organ capable of influencing host health. Recent studies suggest that it should actually be regarded as an “immune system” that can promote health but sometimes initiates disorders such as inflammatory bowel disease, metabolic syndrome, obesity-related disease, diabetes, liver disease and colorectal cancer [210]. Changes in the diet may exert profound effects on the microbiome and are capable of altering the overall bacterial composition. Interactions between the microbiome and the metabolism of dietary components such as phosphatidylcholine and carnitine have been reported to modulate the CVD risk [147]. Moreover, the microbiome could constitute a novel therapeutic opportunity, because in some cases it may be used to detect gut-related diseases earlier than conventional diagnostic workups. In the future, this information could be harnessed to stratify patients more accurately and for more effective treatment [210]. These research fields are still in their infancy. It is therefore critical to clarify the relationship among genetics, diet, microbiome, and health risk.

Finally, useful data are expected to come from systems biology, a highly cross-disciplinary approach to biology research; in turn, biochemical systems biology includes and combines genomics, biochemistry, and molecular biology integrating them with mathematical and computational analysis, engineering practices, and “omics” technologies such as transcriptomics, proteomics and metabolomics [211]. A number of experimental strategies are combining quantitative measurements of cell components (mRNA, proteins, and metabolites) using mathematical and computational models [147,212,213,214,215,216,217,218,219,220,221,222,223,224,225,226,227,228,229,230,231,232]. Such high-throughput technologies are providing a huge amount of functional genomic data that are expected to deliver breakthrough in aging research. According to Özdemir and colleagues, now that the goals and tasks of nutrition science and nutrigenomics have become clearly established, it would be desirable to achieve the integration of four key domains that are naturally connected—agrigenomics, nutrigenomics, nutriproteomics, and nutrimetabolomics—which address complementary issues in relation to individual differences in response to food-related environmental exposure. Although the knowledge and findings of these four omics have still failed to be integrated, they have a very high innovation potential. In the future, personalized nutrition interventions are expected to benefit from the integration of life sciences funding, research, and practice from “farm to clinic to supermarket to society,” and from “genome to proteome to metabolome” [233].

## 4. Molecular Pathological Epidemiology

Epidemiological research typically investigates the factors that are associated with the overall risk of developing certain diseases, including the relationship between exposure and a disease entity in population-based cohorts, whereas pathology research traditionally explores aetiology, development, and histopathological and molecular characteristics to predict prognosis and response to treatment. The merging of the two approaches through the incorporation of molecular pathology into epidemiological research has created a new population health science field that straddles several research areas, which has been designated Molecular Pathological Epidemiology (MPE) [234,235]. Its close relationship to both aetiology and prognosis involves that MPE pursues to gain a greater understanding of how particular exposures influence disease risk through the search and evaluation for molecular pathological markers. Disease processes are influenced by a wide range of exogenous (e.g., acquired genetic and epigenetic alterations, diet, lifestyle, smoking, medications, microorganisms) as well as inherent factors (e.g., germline genetic variations, sex, ethnicity) that induce significant interindividual variability in all phases of the disease process [236]. Compared with conventional approaches, where patients diagnosed with similar symptoms or disease manifestations are assumed to make up a homogeneous group and to share similar causative factors, MPE employs molecular pathological signatures to refine patient categorization and identify subgroups that share more homogenous, to gain insight into disease heterogeneity respect to both aetiologies and pathogenic process. By this approach, the MPE multidisciplinary method explores whether exogenous and endogenous factors are associated with differential molecular signatures and disease subgroups [235,236].

The notion of MPE was first introduced by Ogino and Stampfer in a comment on a case–control study of body mass index (BMI) and the risk of colorectal cancer (CRC) in relation to tumour microsatellite instability (MSI) (MSI-high vs. microsatellite stability [MSS] status) [234,237]. The authors demonstrated that pre-diagnosis BMI was associated with an increased risk of developing CRC, and that the excess risk associated with BMI was limited to MSS tumours [237].

A large number of investigations have subsequently been identified as belonging to MPE. Several of these have identified links between diet, lifestyle, environmental exposure, and alteration of molecular patterns characterized as distinctive features of specific diseases.

Over the past few years, the molecular changes related to the risk of developing CRC have extensively been explored. MPE findings suggest that the effects on outcome of alterations in the WNT signalling pathway and cadherin-associated protein β 1 (CTNNB1 or β-catenin) are modified by BMI and physical activity. In particular, CTNNB1 activation was associated with longer CRC-specific survival and overall survival among obese patients, whereas post-diagnosis physical activity was associated with longer CRC-specific survival only for patients with negative nuclear CTNNB1 status [238]. According to another study, obesity and physical inactivity are associated with a higher risk of CTNNB1-negative CRC, but not of CTNNB1-positive CRC, suggesting that the energy balance and the metabolic state exert effects on a specific carcinogenesis pathway that is less likely to be dependent on WNT/CTNNB1 activation [239]. An important role for BMI has also been found in relation to tumour TP53 mutations, which are key factors in CRC development, and on fatty acid synthase (FASN), which is overexpressed in some colon cancers and in involved in the energy metabolism of fatty acids. MPE data support a dual role for TP53 alterations in cell-cycle deregulation and cell autonomy in relation to the energy balance [240], while FASN-negative and FASN-positive tumours have been reported to be associated with a significantly different CRC risk [241]. Moreover, an excess energy balance may influence the immune and inflammatory status, suggesting an association of BMI with a heightened CRC risk regardless of the level of the lymphocytic response to the tumour [242].

Dietary compounds affect specific pathways related to cancer development. High alcohol consumption increases the CRC risk because one-carbon metabolism triggers a DNA methylation reaction, which affects the CpG island methylator phenotype (CIMP), with tumour epigenetic features modulating the cancer risk [243,244]. Preclinical and epidemiological studies have provided evidence of a protective effect of vitamin D against CRC [245], whereas a higher calcium intake has been associated with a lower risk of developing CRC, especially distal colon cancer; the overall inverse association was linear and did not differ in relation to intake source [246]. Similarly, a high intake of marine ω-3 polyunsaturated fatty acids (ω-3 PUFAs; including eicosapentaenoic acid, docosahexaenoic acid and docosapentaenoic acid) has been documented to have an antineoplastic action. An increased intake of marine ω-3 PUFAs after CRC diagnosis may still confer benefits [247].

A large role for microRNAs has also been documented by MPE studies. The expression level of miR-21 is associated with a worse CRC clinical outcome [248], whereas let-7 family microRNAs suppress adaptive immune responses, contributing to immune evasion by the tumour [249]. Circulating miR-21-5p and miR-126-3p have been shown to play a role as dynamic biomarkers of systemic inflammatory/angiogenic status, and could have an even greater role in managing T2DM [250]. Furthermore, microRNAs have an established potential in the diagnosis and prognosis of several cancers and of pollution exposure [251]. For example, a pool of deregulated circulating and tissue microRNAs with biomarker and therapeutic potential has been identified in malignant mesothelioma, a lethal cancer related to asbestos exposure [252].

MPE findings have also linked leukocyte telomere length (LTL) and genetic variants in the telomerase reverse transcriptase gene region to the risk of pancreatic cancer [253]. LTL shortening is found in a number of ARDs, including T2DM. Analysis of its possible association with mortality was analyzed in this study. Recently, time-dependent mortality risk stratification has allowed demonstrating that T2DM duration and LTL combined with clinical parameters can provide additive prognostic information on mortality risk in these patients [8].

Finally, several lines of MPE evidence have confirmed the link between microbiota and disease. For instance, the abundance of *Fusobacterium nucleatum*, which increases gradually from the rectum to the caecum, reflects the pathogenic influence exerted by the gut microbiota on neoplastic and immune cells, and may promote CRC growth by inhibiting T-cell-mediated immune responses against the tumour. A greater amount of *F. nucleatum* DNA in CRC tissue is associated with shorter survival and may serve as a prognostic biomarker [254,255].

It is highly likely that the MPE data reviewed above can be harnessed to develop new disease prevention and early detection approaches, and that the molecules and pathways identified by MPE research can be used to devise new treatments by targeting altered regulatory mechanisms.

## 5. Conclusions

The notion that aging is a complex, multifactorial process depending on the interaction of genetic and environmental (including lifestyle) factors is widely shared. According to the latest scientific data, human genes are programmed for a lifespan of 120 years. Yet longevity is not merely embedded in the genes; rather, no less than 70% of it needs to be conquered every day by a healthy diet and lifestyle.

The application of integrated omics approaches, together with increasingly detailed nutritional information have vastly improved the exploration and identification of relationships between lifestyle, diet, and health, including the interactions with genetic background and the microbiome, which actively influence them. These advances herald a growing role for nutrigenomics, since the development of personalized nutrition interventions is likely to induce large, appropriate and consistent changes in eating (and other lifestyle) behaviours, thus driving and supporting new prevention and therapeutic strategies, such as microbiome modulation.

Moreover, the evolving field of MPE, which incorporates traditional epidemiology and pathology research, is expected to elucidate how various exposures affect disease onset, transformation and progression. The remit of MPE clearly includes the investigation of the interactive effects of dietary/lifestyle exposures and tumour molecular features on tumour behaviour (prognosis/clinical outcome), so that, ideally, the effects of specific variables can be traced to a specific molecular subtype of cancer [235].
